# Activation of Src-dependent Smad3 signaling mediates the neutrophilic inflammation and oxidative stress in hyperoxia-augmented ventilator-induced lung injury

**DOI:** 10.1186/s12931-015-0275-6

**Published:** 2015-09-16

**Authors:** Li-Fu Li, Chung-Shu Lee, Yung-Yang Liu, Chih-Hao Chang, Chang-Wei Lin, Li-Chung Chiu, Kuo-Chin Kao, Ning-Hung Chen, Cheng-Ta Yang

**Affiliations:** Department of Internal Medicine, Division of Pulmonary and Critical Care Medicine, Chang Gung Memorial Hospital and Chang Gung University, 5 Fu-Hsing Street, Kweishan Taoyuan, 333 Taiwan; Department of Respiratory Therapy, Chang Gung Memorial Hospital, Taoyuan, Taiwan; Chest Department, Taipei Veterans General Hospital, Taipei, Taiwan; Institute of Clinical Medicine, School of Medicine, National Yang-Ming University, Taipei, Taiwan

**Keywords:** Hyperoxia, Matrix metalloproteinase-9, Nicotinamide adenine dinucleotide phosphate oxidase, Smad3, Src, Ventilator-induced lung injury

## Abstract

**Background:**

Mechanical ventilation and concomitant administration of hyperoxia in patients with acute respiratory distress syndrome can damage the alveolar epithelial and capillary endothelial barrier by producing inflammatory cytokines and reactive oxygen species. The Src tyrosine kinase and Smad3 are crucial inflammatory regulators used for ventilator-induced lung injury (VILI). The mechanisms regulating interactions between high-tidal-volume mechanical ventilation, hyperoxia, and acute lung injury (ALI) are unclear. We hypothesized that high-tidal-volume mechanical stretches and hyperoxia augment lung inflammation through upregulation of the Src and Smad3 pathways.

**Methods:**

Wild-type or Src-deficient C57BL/6 mice, aged between 6 and 8 weeks, were exposed to high-tidal-volume (30 mL/kg) ventilation with room air or hyperoxia for 1–4 h after 2-mg/kg Smad3 inhibitor (SIS3) administration. Nonventilated mice were used as control subjects.

**Results:**

We observed that the addition of hyperoxia to high-tidal-volume mechanical ventilation further induced microvascular permeability, neutrophil infiltration, macrophage inflammatory protein-2 and matrix metalloproteinase-9 (MMP-9) production, malondialdehyde, nicotinamide adenine dinucleotide phosphate oxidase activity, MMP-9 mRNA expression, hypoxemia, and Src and Smad3 activation (*P* < 0.05). Hyperoxia-induced augmentation of VILI was attenuated in Src-deficient mice and mice with pharmacological inhibition of Smad3 activity by SIS3 (*P* < 0.05). Mechanical ventilation of Src-deficient mice with hyperoxia further reduced the activation of Smad3.

**Conclusions:**

Our data suggest that hyperoxia-increased high-tidal-volume ventilation-induced ALI partially depends on the Src and Smad3 pathways.

## Introduction

Acute respiratory distress syndrome (ARDS) is a disorder of acute hypoxemic respiratory failure marked by increased microvascular permeability and capillary leak because of disruption of the epithelial-endothelial barrier, which consists of alveolar epithelium, capillary endothelium, and extracellular matrix (ECM) [1–3]. Mechanical ventilation with high levels of oxygen has been employed for life support in patients with ARDS; however, the potential for hyperinflation of the intact parts of the lungs and production of reactive oxygen species (ROS) is high [4–6]. Ventilator-induced lung injury (VILI) is characterized by noncardiogenic pulmonary edema, the release of cytokines and chemokines leading to recruitments of neutrophils [7–11]. Previous studies have demonstrated that high tidal volume (V_T_) mechanical ventilation may upregulate Src, Smads, and the production of inflammatory cytokines, including tumor necrosis factor-α (TNF-α), macrophage inflammatory protein-2 (MIP-2), and matrix metalloproteinase-9 (MMP-9) in acute lung injury (ALI) [4, 7–13]. However, the mechanisms regulating the interactions between mechanical ventilation and these inflammatory reactions remain unclear.

Prolonged exposure to hyperoxia causes the generation of ROS, including superoxide, hydrogen peroxide, and hydroxyl radical, which may overwhelm the protection of antioxidants in ALI [14]. High-tidal-volume ventilation with hyperoxia has been shown in rat models to induce neutrophil migration into the alveoli and is dependent on MIP-2 production, a chemoattractant belonging to the cysteine-amino-cysteine family of cytokine [4, 6]. MMP-9, a 92-kDa proteolytic enzyme released from activated neutrophils, is required for their transendothelial migration [15, 16]. MMP-9 can cleave gelatin and type IV collagen present in basement membranes and causes disruption of the pulmonary alveolar-capillary integrity, contributing to high-permeability lung edema in ALI [15–17]. Moreover, Foda et al., in a rat model of high-tidal-volume mechanical ventilation, demonstrated that MMPs are crucial for the development of VILI [18].

The Src family is composed of non-receptor tyrosine kinases, which regulate cell proliferation, migration, apoptosis, and ECM adhesion functions [7]. These protein kinases are expressed by macrophages, neutrophils, endothelial cells, alveolar epithelial cells, and fibroblasts in the lung [12]. In addition to their roles in inducing fibrogenesis, Src and Smad2/3 phosphorylation are increased in mice after endotoxin treatment or in bronchial epithelial cells in response to endoplasmic reticulum stress [19, 20]. Previous studies of hyperoxia-mediated ALI have demonstrated the involvement of the Src pathway in the regulation of leukocyte infiltration, fibrin deposition, activation of nicotinamide adenine dinucleotide phosphate (NADPH) oxidase (NOX), and MIP-2 and MMP-9 production [21–24]. Furthermore, recent studies of sepsis-induced ALI in rats have revealed that pharmacologic inhibition of transforming growth factor-β1 (TGF-β1) and the Smad3 signaling pathways substantially ameliorated epithelial and endothelial microvascular permeability [3, 13, 25]. Given the absence of a proven effective treatment for ALI and subsequent lung fibrogenesis, Src and Smad3 inhibition might provide an attractive target in the deteriorating process of patients with ARDS.

Using this murine model of hyperoxia-mediated VILI, we compared the relationships among high-tidal-volume ventilation with or without hyperoxia, the correlation of Src and Smad3 activations to neutrophil influx, oxidative stress, severity of lung injury, and MIP-2 and MMP-9 production by using a specific inhibitor of Smad3 (SIS3) and Src-deficient mice. We hypothesized that the addition of hyperoxia to high-tidal-volume mechanical ventilation would increase neutrophil infiltration, NADPH oxidase expression, and MIP-2 and MMP-9 production secondary to activating the Src and Smad3 pathways.

## Materials and methods

### Ethics of experimental animals

Wild-type or Src-deficient C57BL/6 mice, weighing between 20 and 25 g, aged between 6 and 8 weeks, were obtained from Jackson Laboratories (Bar Harbor, ME) and National Laboratory Animal Center (Taipei, Taiwan) [8, 26]. Src-deficient mice were generated by target gene disruption as previously described [8]. The experiments were performed in accordance with the National Institutes of Health Guidelines on the Use of Laboratory Animals and approved by the Institutional Animal Care and Use Committee of Chang Gung Memorial Hospital (Permit number: 2011093005).

### Experimental groups

Animals were randomly distributed into 9 groups in each experiment: group 1, control, nonventilated wild-type mice with room air; group 2, control, nonventilated wild-type mice with hyperoxia; group 3, V_T_ 30 mL/kg wild-type mice with room air; group 4, V_T_ 30 mL/kg wild-type mice with hyperoxia; group 5, V_T_ 30 mL/kg wild-type mice after SIS3 administration with hyperoxia; group 6, V_T_ 30 mL/kg Src^+/−^ mice with hyperoxia; group 7, control, nonventilated wild-type Src^+/−^ mice with room air; group 8, V_T_ 30 mL/kg wild-type mice after SIS3 administration with room air; and group 9, V_T_ 30 mL/kg Src^+/−^ mice with room air. In group 1–6, three mice underwent transmission electron microscopy and five mice underwent measurement for bronchoalveolar lavage (BAL) fluid total protein, cell counts, Evans blue dye (EBD) assay, gelatin zymography, histology, immunohistochemistry assay, lung edema, myeloperoxidase (MPO), malondialdehyde (MDA), total antioxidant capacity, NADPH oxidase assay, real-time polymerase chain reaction (PCR), Western blot, and MIP-2 and MMP-9. In group 7–9, four mice underwent measurement for EBD assay and lung edema.

### Ventilator protocol

We used our established mouse model of VILI, as previously described [4, 6]. Mean arterial pressure was monitored every hour during mechanical ventilation by using the data acquisition system (iWorx 214, iWorx Systems, Inc., Dover, NH) connected to a 0.61-mm outer diameter (0.28-mm inner diameter) polyethylene catheter ending in the common carotid artery. Our previous work has shown that activation of Src and increased mRNA expression of what occurred around 1 h of lung stretch, whereas changes in cytokine production and neutrophil infiltration occur later [8, 27]. One hour of mechanical ventilation was employed for gelatin zymography, real-time PCR, and Western blot analyses, and 4 h was applied for MIP-2 and MMP-9 production, BAL fluid total protein, cell counts, lung edema, EBD, and histologic staining analyses, transmission electron microscopy.

### SIS3 administration

SIS3 is a Smad3 inhibitor (2-mg/kg; Calbiochem, La Jolla, CA), and was given intraperitoneally 1 h before mechanical ventilation, based on previous *in vivo* study that showed 2-mg/kg SIS3 inhibited Smad3 activity and lung injury [28].

### Measurement of MIP-2 and MMP-9

At the end of the study period, the lungs were lavaged via tracheostomy with 20-gauge angiocatheter 3 times with 0.6 mL of 0.9 % normal saline. The effluents were pooled and centrifuged at 310 × g for 10 min. Supernatants were frozen at −80 °C for further analysis of cytokines. MIP-2 with a lower detection limit of 1 pg/mL and MMP-9 with a lower detection limit of 14 pg/mL were measured in bronchoalveolar lavage fluid using a commercially available immunoassay kit containing primary polyclonal anti-mouse antibody that was cross-reactive with rat and mouse MIP-2 and MMP-9 (Biosource International, Camarillo, CA). Each sample was run in duplicate according to the manufacturer’s instructions.

### Gelatin zymography

The activity of MMP-9 in lung tissues was measured using gelatin zymography, as previously described [10]. Briefly, the lung tissue homogenates were matched for protein concentration and resolved on a 10 % bis-acrylamide gel copolymerized with 2-mg/mL of gelatin. After electrophoresis, gels were rinsed in 2.5 % Triton × −100 twice for 20 min to remove sodium dodecyl sulfate. The gels were then incubated in incubation buffer (50 mM Tris–HCl, 10 mM CaCl2, 150 mM NaCl, and 0.05 % NaN3) at 37 °C for 24 h. The gels were stained in 0.05 % Coomassie Brilliant Blue G-250 (Sigma, St. Louis, MO) in a mixture of methanol: glacial acetic acid: water (2.5:1:6.5), v: v) and destained in aqueous 4 % methanol: 8 % glacial acetic acid (*v: v*). Developed gels were then scanned through densitometry.

### Measurement of malondialdehyde and total antioxidant capacity

The lungs were homogenized in phosphate buffered saline with or without containing butylated hydroxytoluene for MDA and total antioxidant capacity, respectively. The malondialdehyde and total antioxidant capacity in the protein extracts were measured using the Oxiselect TBARS assay kit containing thiobarbituric acid reactive substances and Oxiselect total antioxidant assay kit containing uric acid (Cell Biolabs, San Diego, CA). Each sample was run in duplicate and expressed as μmole/g protein for MDA and μmole Copper reducing equivalents/g protein for total antioxidant capacity according to the manufacturer’s instructions.

### Immunoblot analysis

The lungs were homogenized in 3 mL of lysis buffer (20 mM HEPES pH 7.4, 1 % Triton × -100, 10 % glycerol, 2 mM ethylene glycol-bis (β-aminoethyl ether)-N, N, N’, N’-tetraacetic acid, 50 μM β-glycerophosphate, 1 mM sodium orthovanadate, 1 mM dithiotreitol, 400 μM aprotinin, and 400 μM phenylmethylsulfonyl fluoride), transferred to eppendorf tubes and placed on ice for 15 min. The above chemicals were purchased from Sigma-Aldrich (St. Louis, MO). Tubes were centrifuged at 15,350 × g for 10 min at 4 °C and supernatant was flash-frozen. Crude cell lysates were matched for protein concentration, resolved on a 10 % bis-acrylamide gel, and electrotransferred to Immobilon-P membranes (Millipore Corp., Bedford, MA). For assay of Src phosphorylation and Src, Smad3 phosphorylation and Smad3, NOX2, and glyceraldehydes-phosphate dehydrogenase (GAPDH) total protein expression, Western blot analyses were performed with phospho-Src, Src, phospho-Smad3, Smad3, NOX2, and GAPDH antibodies (New England BioLabs, Beverly, MA). Blots were developed by enhanced chemiluminescence (NEN Life Science Products, Boston, MA).

### Immunohistochemistry

The lungs were paraffin embedded, sliced at 4 μm, deparaffinized, antigen unmasked in 10 mM sodium citrate (pH 6.0), incubated with rabbit phospho-Src and phosopho-Smad3 primary antibody (1:100; New England BioLabs, Beverly, MA), and biotinylated goat anti-rabbit secondary antibody (1:100) according to the manufacturer’s instruction for an immunohistochemical kit (Santa Cruz Biotechnology, Santa Cruz, CA). The specimens were further conjugated with horseradish peroxidase-streptoavidin complex, detected with a diaminobenzidine (DAB) substrate mixture, and counterstained by hematoxylin. A dark-brown DAB signal, identified by arrows, indicated positive staining of phospho-Src and phospho-Smad3 of epithelial cells, whereas shades of light blue signified nonreactive cells.

### Real-time polymerase chain reaction

For isolating total RNA, the lung tissues were homogenized in TRIzol reagents (Invitrogen Corporation, Carlsbad, CA) according to the manufacturer’s instructions. Total RNA (1 μg) was reverse transcribed by using a GeneAmp PCR system 9600 (PerkinElmer, Life Sciences, Inc., Boston, MA), as previously described [6]. The following primers were used for real-time PCR: MMP-9, forward primer 5’- CCTACTGCGGGCTCTTCTGA −3’ and reverse primer 5’- CCCTGTAATGGGCTTCCTCT −3’ and GAPDH as internal control, forward primer 5’- AATGCATCCTGCACCACC AA −3’ and reverse primer 5’- GTAGCCATATTCATTGTCATA −3’ (Integrated DNA Technologies, Inc., Coralville, IA) [29]. All quantitative PCR reactions using SYBR Master Mix were performed on an ABI Prism 7000 sequence detector PCR system (Applied Biosystems, Foster City, CA). All PCR reactions were performed in duplicate and heated to 94 °C for 5 min followed by 30 cycles of denaturation at 94 °C for 30 s, annealing at 59 °C for 45 s, and extension at 72 °C for 1 min. The standard curves (cycle threshold values versus template concentration) were prepared for each target gene and for the internal control (GAPDH) in each sample.

Analysis of BAL fluid total protein, cell counts, EBD analysis, histopathologic grading of VILI, NADPH oxidase assay, lung edema MPO assay, transmission electron microscopy, and ventilator protocol were performed as previously described [6, 8].

### Statistical evaluation

The gelatin zymogrphy and Western blots were quantitated using a National Institutes of Health (NIH) image analyzer Image J 1.27z (National Institutes of Health, Bethesda, MD) and presented as arbitrary units. Values were expressed as the mean ± SD from at least 5 separate experiments. The data of BAL fluid total protein, cell counts, EBD assay, lung wet-to-dry weight ratio, MIP-2 and MMP-9, MMP-9 mRNA, MPO, NADP^+^/NADPH, histopathologic assay, and oxygenation were analyzed using Statview 5.0 (Abascus Concepts Inc. Cary, NC; SAS Institute, Inc.). All results of gelatin zymography, real-time PCR, and Western blots were normalized to the nonventilated control wild-type mice with room air. ANOVA was used to assess the statistical significance of the differences, followed by multiple comparisons with a Scheffe’s test, and a *P* value < 0.05 was considered statistically significant.

## Results

### Suppression of the effects of hyperoxia on VILI by SIS3 and Src-deficient mice

**Fig. 1:**
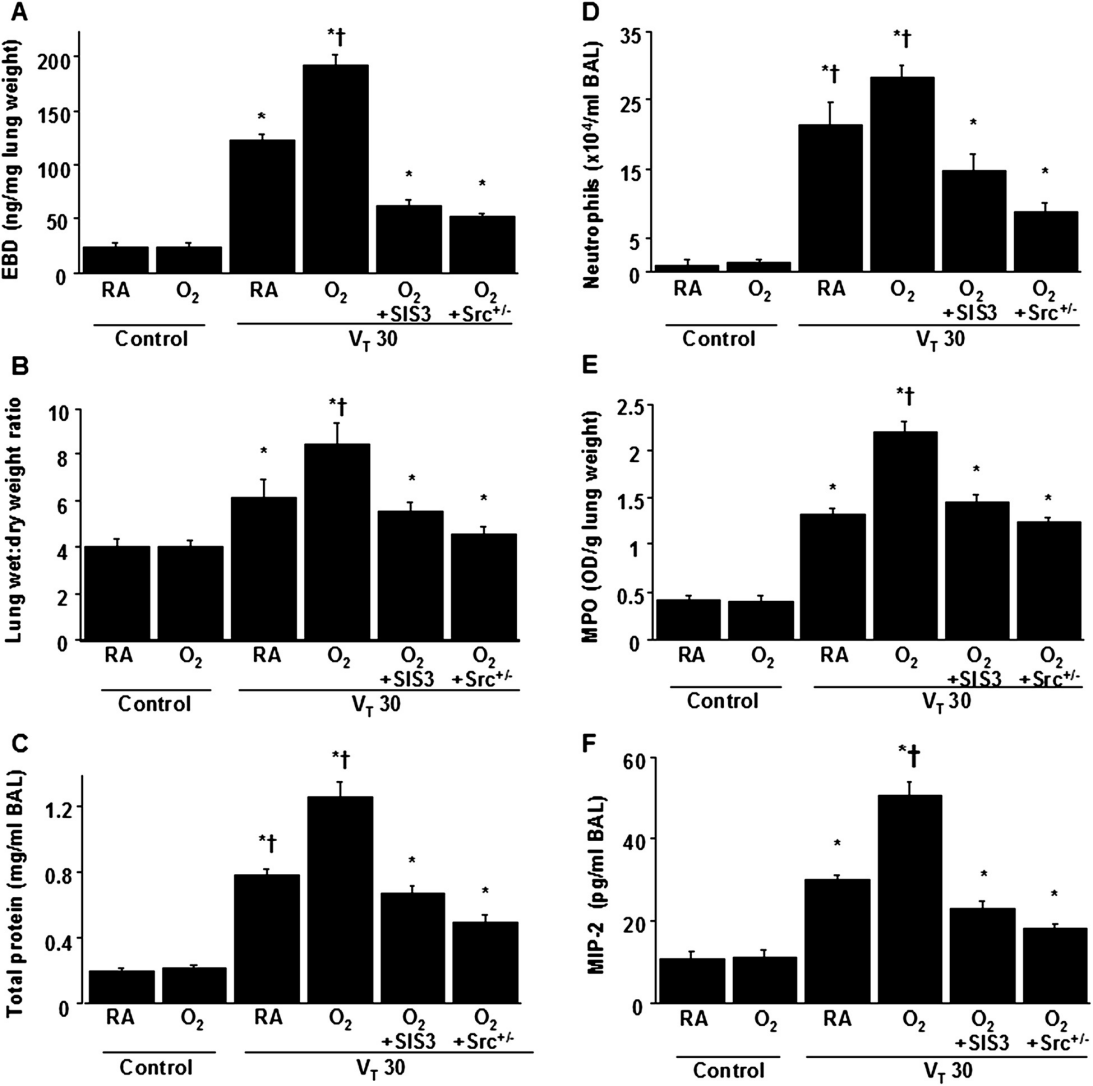
SIS3 and Src-deficient mice reduced hyperoxia-augmented lung stretch-induced ventilator-induced lung injury. The effects of administering SIS3 or Src heterozygous knockout on **a** EBD analysis, **b** lung wet-to-dry-weight ratio, **c** BAL fluid total protein, **d** neutrophil counts, **e** MPO activity, and **f** MIP-2 secretion in BAL fluid were from the lungs of nonventilated control mice and those subjected to a tidal volume of 30 mL/kg (V_T_ 30) for 4 h with room air or hyperoxia (*n* = 5 per group). SIS3 2-mg/kg was administered intraperitoneally 1 h before ventilation. *P < 0.05 versus the nonventilated control mice with room air; **†** *P* < 0.05 versus all other groups. BAL = bronchoalveolar lavage; EBD = Evans blue dye; MIP-2 = macrophage inflammatory protein-2; MPO = myeloperoxidase; O_2_ = mice with hyperoxia; RA = mice with room air; SIS3 = specific inhibitor of Smad3; Src^+/− =^ Src-deficient mice

**Fig. 2:**
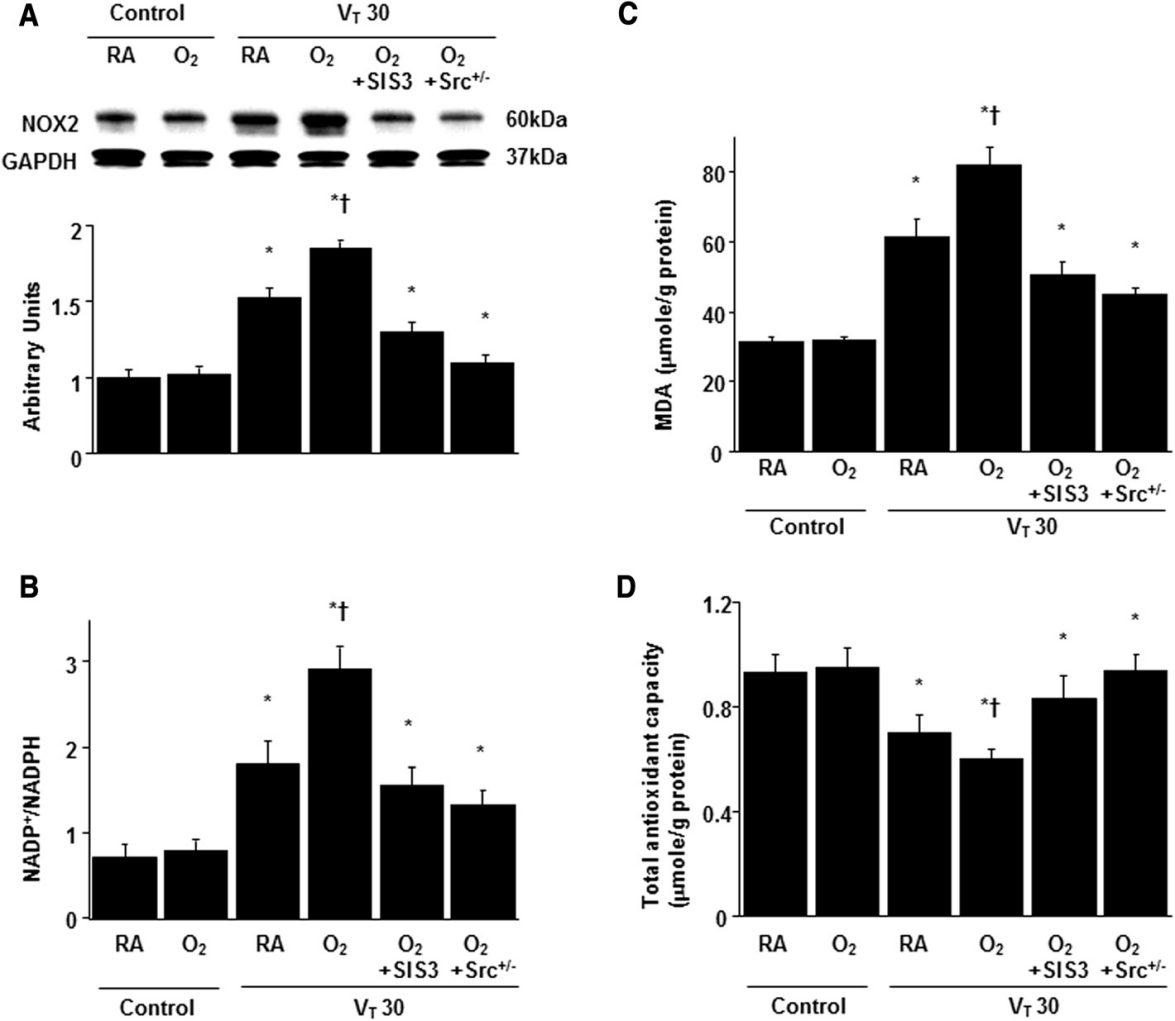
SIS3 and Src-deficient mice inhibited hyperoxia-augmented lung stretch-mediated oxidative stress. **a** A Western blot was performed using an antibody that recognizes NOX2 expression and an antibody that recognizes GAPDH expression from the lungs of nonventilated control mice and those subjected to a tidal volume of 30 mL/kg for 1 h with room air or hyperoxia. Arbitrary units were expressed as the ratio of NOX2 to GAPDH (*n* = 5 per group). **b** NADP^+^-to-NADPH ratio, **c** MDA and **d** total antioxidant capacity were from the lungs of nonventilated control mice and those subjected to a tidal volume of 30 mL/kg for 4 h with room air or hyperoxia (*n* = 5 per group). SIS3 2-mg/kg was given intraperitoneally 1 h before ventilation. *P < 0.05 versus the nonventilated control mice with room air; **†** *P* < 0.05 versus all other groups. GAPDH = glyceraldehydes-phosphate dehydrogenase; MDA = malondialdehyde; NADP^+^ = nicotinamide adenine dinucleotide phosphate; NADPH = reduced NADP^+^; NOX2 = NADPH oxidase 2

High-tidal-volume (30 mL/kg) ventilation with room air or hyperoxia for 4 h was employed to induce VILI in mice. The injurious effects of hyperinflation and inhibitory effects of intraperitoneally delivered SIS3 were identified by measuring lung EBD leak, wet-to-dry weight ratio, and BAL fluid total protein and shown in Fig. 1. The physiological conditions at the beginning and end of mechanical ventilation are shown in Table 1. The stable hemodynamic statuses of mice were maintained by monitoring their mean artery pressures. The lung EBD leak, wet-to-dry weight ratio, and BAL fluid total protein were measured to determine the effects of high-tidal-volume ventilation with and without hyperoxia on microvascular permeability and lung edema in VILI (Fig. 1a, b, c). Furthermore, neutrophil counts, MPO activity, and MIP-2 protein production were examined to quantitate neutrophils, the main inflammatory cells and a potential source of ROS, and chemoattractants associated with VILI and concomitant hyperoxia (Fig. 1c, d, e). Additionally, the expression of an oxidant-generating enzyme, NOX2, the NADP^+^-to-NADPH ratio, the levels of oxidant loads and antioxidant capacity were measured to determine the level of oxidative stress in hyperoxia-induced ALI (Fig. 2). Increased levels of EBD, wet-to-dry weight ratio, BAL fluid total protein, neutrophil counts, MPO, MIP-2 protein, NADP^+^-to-NADPH ratio, activity of NOX2, and MDA but reduced production of total antioxidant capacity were observed in mice subjected to a tidal volume of 30 mL/kg with hyperoxia compared with those subjected to a tidal volume of 30 mL/kg with room air and nonventilated control mice. The increases of lung inflammation with a tidal volume of 30 mL/kg mechanical ventilation and hyperoxia were substantially suppressed by treatment with SIS3 or with Src-deficient mice. Reduced production of MDA, which is an aldehydic secondary product of lipid peroxidation, but increased production of total antioxidant capacity, was found after pharmacological inhibition with SIS3 or with Src-deficient mice. There were no statistically significant differences between EBD and wet-to-dry ratio in wild-type or Src-deficient non-ventilated control mice with room air, in wild-type mice subjected to a tidal volume of 30 mL/kg pretreated with SIS3 with or without hyperoxia, and in Src-deficient mice subjected to a tidal volume of 30 mL/kg with or without hyperoxia (the levels of EBD: control, nonventilated wild-type mice with room air = 23.6 ± 1.7 ng/mg lung weight versus control, nonventilated Src-deficient mice with room air 22.3 ± 1.2 ng/mg lung weight, *P* = 0.36; V_T_ 30 mL/kg wild-type mice after SIS3 administration with room air = 61.7 ± 2.6 ng/mg lung weight versus V_T_ 30 mL/kg wild-type mice after SIS3 administration with hyperoxia = 64.1 ± 2.3 ng/mg lung weight, *P* = 0.29; V_T_ 30 mL/kg Src^+/−^ mice with room air = 51.2 ± 3.2 ng/mg lung weight versus V_T_ 30 mL/kg Src^+/−^ mice with hyperoxia = 52.9 ± 2.5 ng/mg lung weight, *P* = 0.51; wet-to-dry weight ratio: control, nonventilated wild-type mice with room air = 4.2 ± 0.2 versus control, nonventilated Src-deficient mice with room air = 4.0 ± 0.1, *P* = 0.24; V_T_ 30 mL/kg wild-type mice after SIS3 administration with room air = 5.0 ± 0.4 versus V_T_ 30 mL/kg wild-type mice after SIS3 administration with hyperoxia = 5.3 ± 0.3, *P* = 0.71; V_T_ 30 mL/kg Src^+/−^ mice with room air = 4.5 ± 0.3 versus V_T_ 30 mL/kg Src^+/−^ mice with hyperoxia = 4.7 ± 0.2, *P* = 0.49).

**Table 1:** Physiologic conditions at the beginning and end of ventilation

	Control, wild-type, Room air	Control, wild-type, Hyperoxia	V_T_ 30 mL/kg, wild-type, Room air	V_T_ 30 mL/kg, wild-type, Hyperoxia	V_T_ 30 mL/kg, wild-type, Hyperoxia with SIS3	V_T_ 30 mL/kg, wild-type, Hyperoxia with Src^+/−^
PH	7.42 ± 0.04	7.39 ± 0.03	7.38 ± 0.05	7.34 ± 0.03	7.39 ± 0.02	7.37 ± 0.05
PaO_2_ (mmHg)	97.6 ± 0.5	426.3 ± 4.9	86.5 ± 0.6*	387.9 ± 4.0*^,^**	406.8 ± 3.9*	415.6 ± 4.2*
PaCO_2_ (mmHg)	39.6 ± 0.4	39.5 ± 0.3	36.9 ± 1.3	43.6 ± 1.5	41.9 ± 1.8	41.2 ± 1.7
MAP (mmHg)						
Start	85.9 ± 1.6	86.3 ± 1.5	85.2 ± 1.4	84.9 ± 2.1	85.1 ± 2.7	84.8 ± 1.8
End	85.1 ± 0.7	85.4 ± 0.8	77.6 ± 2.7*	75.2 ± 2.5*^,^**	78.5 ± 2.0*	79.8 ± 2.3*
PIP, mm Hg						
Start			24.3 ± 1.2	25.2 ± 1.3	24.8 ± 1.5	25.1 ± 1.3
End			27.6 ± 1.4	28.7 ± 1.8	28.0 ± 1.3	27.9 ± 1.2

At the end of the study period, we obtained data of arterial blood gases and mean arterial pressures from the nonventilated control mice and mice subjected to a tidal volume of 30 mL/kg for 4 h (n = 10 per group). We maintained the normovolemic statuses of mice by monitoring the mean artery pressure. Data are presented as mean ± SD

Time points for measurements were determined according to our previous findings that mediator activation occurs early in ventilator-induce lung injury and neutrophil infiltration occurs later [8, 27]. Because no differences were observed between wild-type or Src-deficient non-ventilated control mice with room air, wild-type mice subjected to a tidal volume of 30 mL/kg pretreated with SIS3 with or without hyperoxia, Src-deficient mice subjected to a tidal volume of 30 mL/kg with or without hyperoxia, the data were combined. The physiological data on the nonventilated control groups were similar during the experiment and were used as ventilation start data

MA*P* = mean arterial pressure; PIP = peak inspiratory pressure; SIS3 = specific inhibitor of Smad3; Src^+/−^ = Src-deficient mice; V_T_ = tidal volume

*indicates that *P* < 0.05 when compared to the nonventilated control mice with room air and **indicates that *P* < 0.05 when compared to all other groups

### Inhibition of the effects of hyperoxia on lung stretch-induced activation of MMP-9 by SIS3 and in Src-deficient mice

**Fig. 3:**
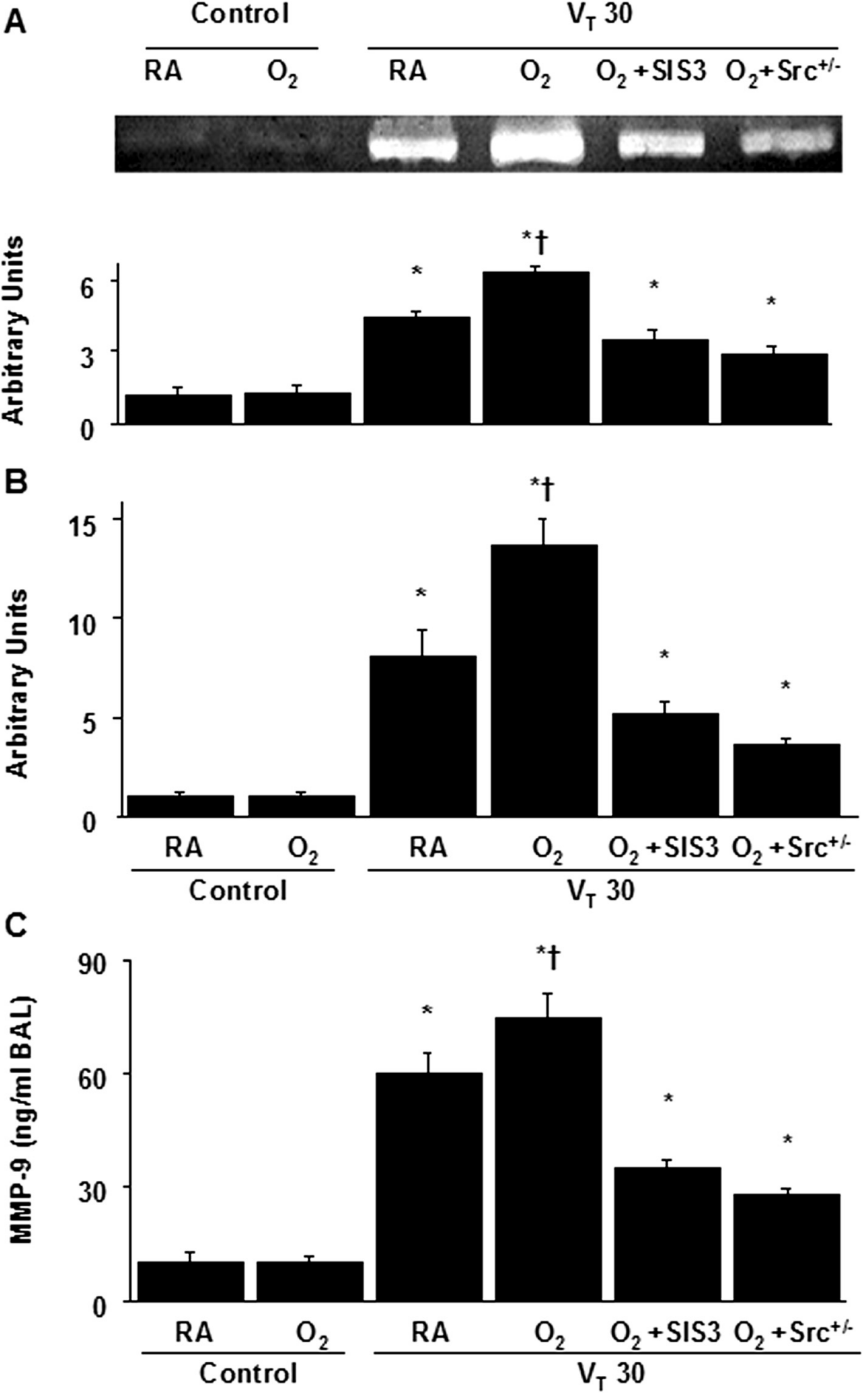
SIS3 and Src-deficient mice attenuated hyperoxia-augmented high-tidal-volume-induced MMP-9 activation, MMP-9 mRNA expression, and MMP-9 production. **a** Gelatin zymography of MMP-9 activity and **b** real-time polymerase chain reaction of MMP-9 mRNA expression were from the lungs of nonventilated control mice and those subjected to a tidal volume of 30 mL/kg for 1 h with room air or hyperoxia. Arbitrary units were the results of MMP-9 gelatinase activity normalized to the nonventilated control wild-type mice with room air and the ratio of MMP-9 mRNA to GAPDH (*n* = 5 per group). **c** MMP-9 protein production in BAL fluid were from the lungs of nonventilated control mice and those subjected to a tidal volume of 30 mL/kg for 4 h with room air or hyperoxia (*n* = 5 per group). SIS3 2-mg/kg was administered intraperitoneally 1 h before ventilation. *P < 0.05 versus the nonventilated control mice with room air; **†** *P* < 0.05 versus all other groups. MMP-9 = matrix metalloproteinase-9

MMP-9 has been shown to play a crucial role in modulating neutrophil-related ALI [9, 10]. The gelatinase activity of MMP-9, MMP-9 mRNA expression associated with chemotactic factors for neutrophils, and MMP-9 protein production in mice subjected to a tidal volume of 30 mL/kg were measured to determine the effects of MMP-9 in VILI with hyperoxia. The elevation of stretch-induced MMP activity using hyperoxia was substantially lowered in Src-deficient mice and pharmacologic inhibition with SIS3 (Fig. 3).

### Reduction of the effects of hyperoxia on mechanical ventilation-induced expression of Smad3 by Src-deficient mice and SIS3

**Fig. 4:**
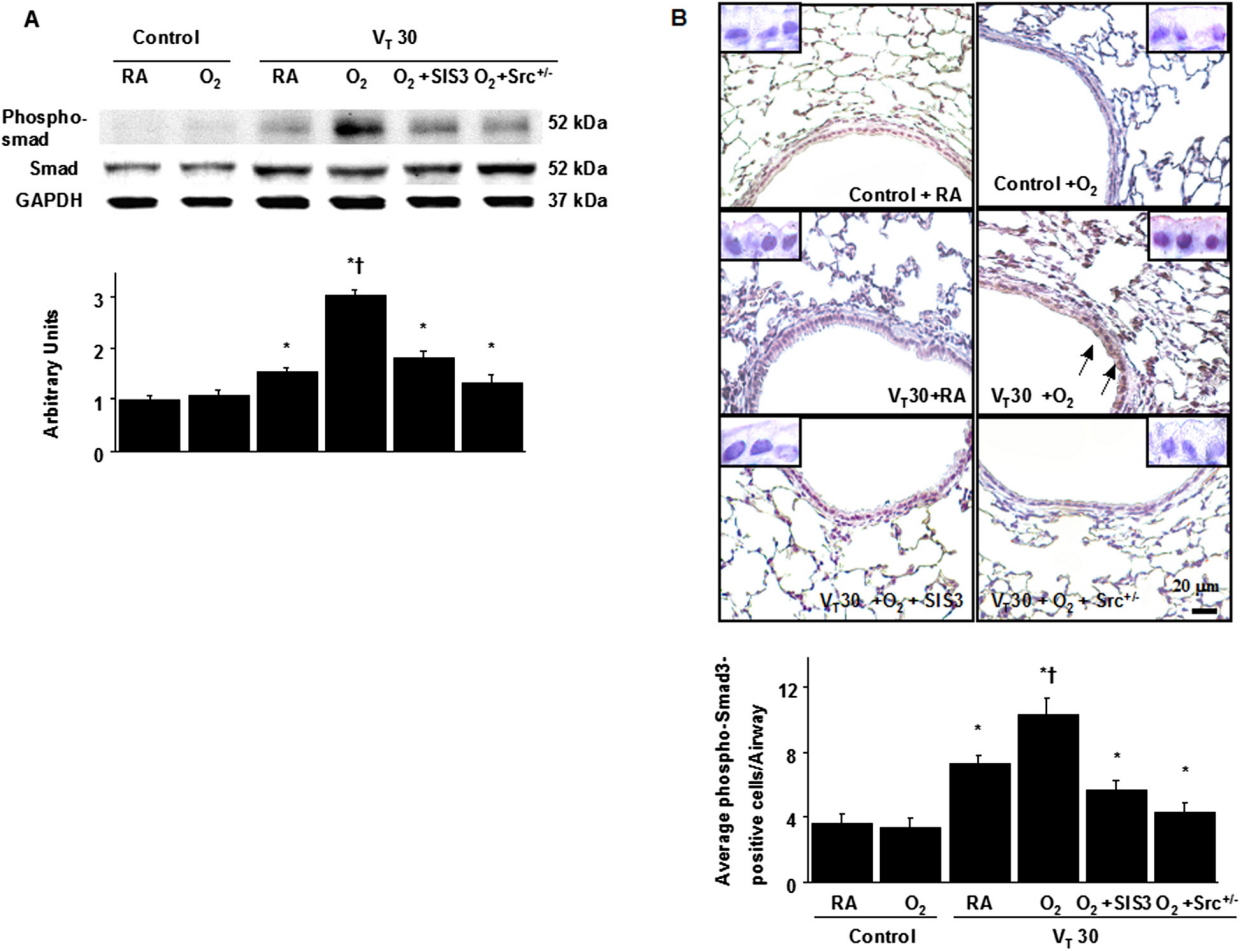
SIS3 and Src-deficient mice suppressed hyperoxia-augmented lung stretch-mediated Smad3 phosphorylation. **a** A Western blot was conducted by using an antibody that recognizes the phosphorylated Smad3 expression and an antibody that recognizes total Smad3 expression were from the lungs of nonventilated control mice and those subjected to a tidal volume of 30 mL/kg for 1 h with room air or hyperoxia. Arbitrary units were expressed as the ratio of phospho-Smad3 to Smad3 (*n* = 5 per group). **b** Representative micrographs (×400) with phosphorylated Smad3 staining of paraffin lung sections and quantification were from the lungs of nonventilated control mice and those subjected to a tidal volume of 30 mL/kg for 4 h with room air or hyperoxia (*n* = 5 per group). SIS3 2-mg/kg was given intraperitoneally 1 h before ventilation. A dark-brown diaminobenzidine signal identified by arrows indicates positive staining for phospho-Smad3 in the lung epithelium or interstitial, whereas shades of bluish tan signify nonreactive cells. *P < 0.05 versus the nonventilated control mice with room air; **†** *P* < 0.05 versus all other groups. Scale bars represent 20 μm

Western blot analysis and immunohistochemical staining were performed to identify Smad3 expression and the cell types involved in the VILI model with hyperoxia (Fig. 4). Western blot analyses revealed increased Smad3 phosphorylation in mice subjected to a tidal volume of 30 mL/kg with hyperoxia compared with those subjected to a tidal volume of 30 mL/kg with room air and nonventilated control mice. Additionally, Western blot analyses showed that Src knockout and SIS3 attenuated the high-tidal-volume-induced phospho-Smad activation by 56 % (*P* < 0.05) and 40 % (*P* < 0.05), respectively (Fig. 4a). The expression of total nonphosphorylated proteins of Smad3 did not change significantly. Consistent with the Western blot results, the positive immunohistochemical staining for phospho-smad3 in the lung epithelium of mice after a tidal volume of 30 mL/kg mechanical ventilation with hyperoxia was significantly attenuated by Src-deficient mice and SIS3 (Fig. 4b). These data suggested the involvement of the Smad3 pathway in regulating VILI.

### The effects of hyperoxia on the mechanical ventilation-induced Src pathway were diminished in Src-deficient mice

**Fig. 5:**
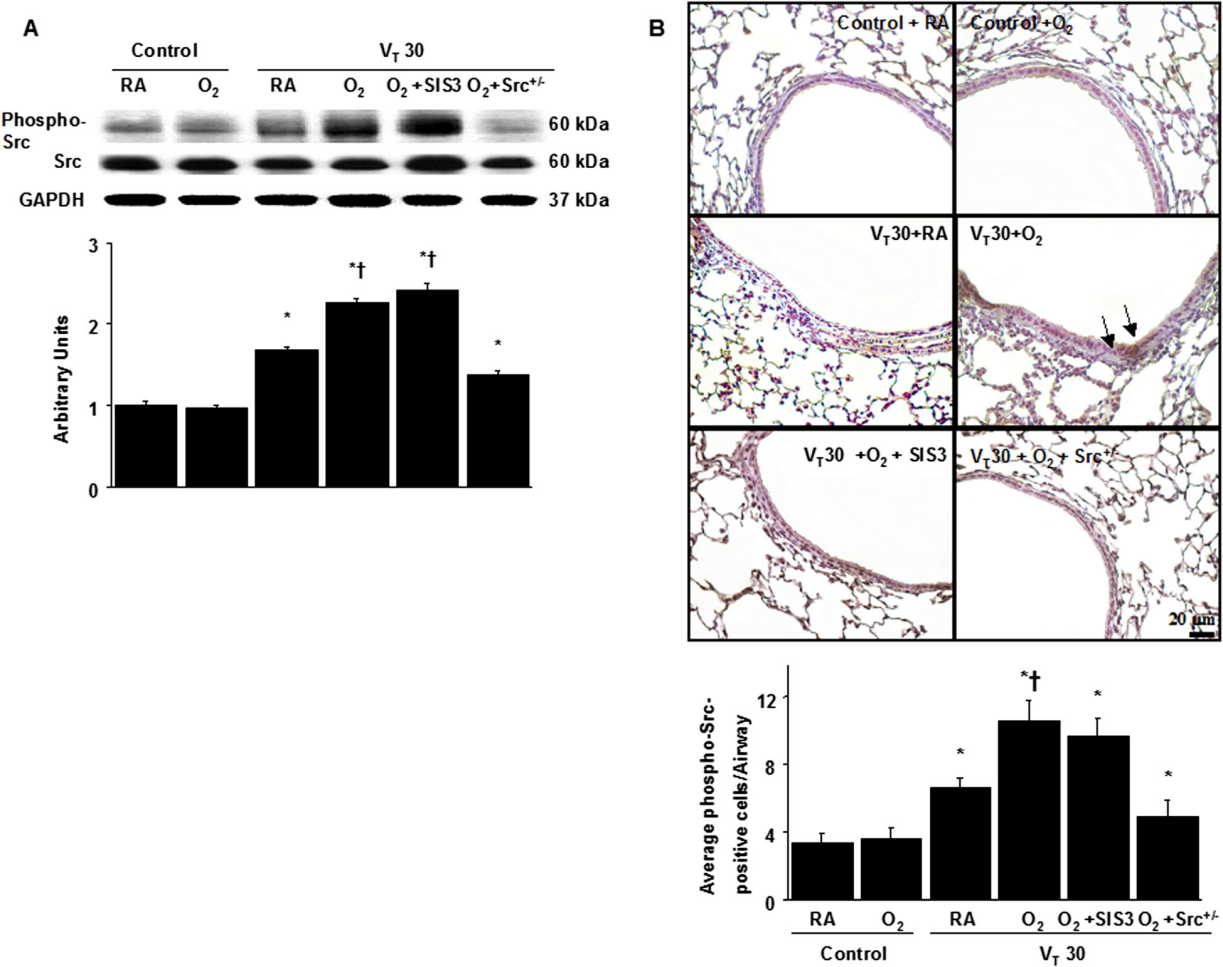
Src-deficient mice abrogated hyperoxia-augmented lung stretch-induced Src phosphorylation. **a** A Western blot was conducted by using an antibody that recognizes the phosphorylated Src expression and an antibody that recognizes total Src expression were from the lungs of nonventilated control mice and those subjected to a tidal volume of 30 mL/kg for 1 h with room air or hyperoxia. Arbitrary units were expressed as the ratio of phospho-Src to Src (*n* = 5 per group). **b** Representative micrographs (×400) with phosphorylated Src staining of paraffin lung sections and quantification were from the lungs of nonventilated control mice and those subjected to a tidal volume of 30 mL/kg for 4 h with room air or hyperoxia (*n* = 5 per group). SIS3 2-mg/kg was administered intraperitoneally 1 h before ventilation. A dark-brown diaminobenzidine signal identified by arrows indicates positive staining for phospho-Src in the lung epithelium or interstitial, whereas shades of bluish tan signify nonreactive cells. *P < 0.05 versus the nonventilated control mice with room air; **†** *P* < 0.05 versus Src-deficient mice. Scale bars represent 20 μm

To further examine the roles of Src and Smad3 in hyperoxia-related ALI, Western blot analysis and immunohistochemical assay were performed to identify Src expression (Fig. 5). Western blot analyses revealed increased Src phosphorylation in mice subjected to a tidal volume of 30 mL/kg with hyperoxia compared with those subjected to a tidal volume of 30 mL/kg with room air and nonventilated control mice. Moreover, high-tidal-volume ventilation plus hyperoxia-induced phospho-Src activation was attenuated by Src-deficient mice but not inhibited by SIS3 (Fig. 5a). The expression of total nonphosphorylated proteins of Src did not change significantly. Additionally, the airway epithelial cells positively stained for phospho-Src were substantially increased in mice subjected to a tidal volume of 30 mL/kg and concurrent administration of hyperoxia compared with those subjected to a tidal volume of 30 mL/kg with room air and nonventilated control mice (Fig. 5b). Consistent with the Western blot results, the increases of Src phosphorylation after mechanical ventilation were substantially lowered in Src-deficient mice but not inhibited by SIS3, suggesting that Src was the upstream regulator of Smad3 signaling involved in both VILI and hyperoxia.

### Inhibition of hyperoxia-augmented stretch-induced lung injuries and improved oxygenation by Src-deficient mice and SIS3

**Fig. 6:**
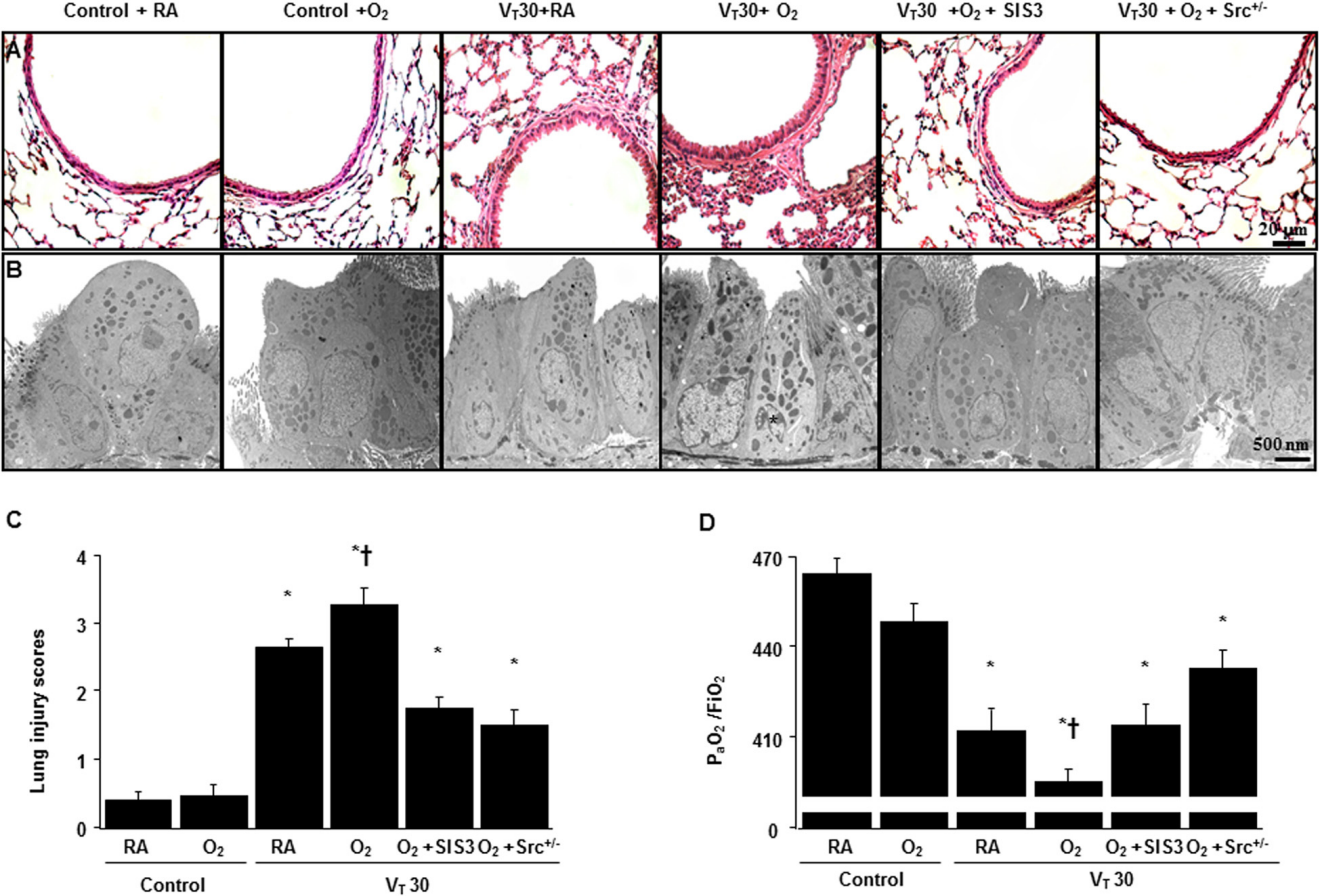
SIS3 and Src-deficient mice ameliorated hyperoxia-augmented lung stretch-mediated lung damage and hypoxemia. **a** Histological examination (×400, *n* = 5 per group), **b** Transmission electron microscopy (×6000, *n* = 3 per group), **c** lung injury scores, and **d** PaO_2_/FiO_2_ were from the nonventilated control mice and those subjected to a tidal volume of 30 mL/kg for 4 h with room air or hyperoxia (*n* = 5 per group). SIS3 2-mg/kg was given intraperitoneally 1 h before ventilation. Apoptotic cells are identified by asterisks. Highly condensed and fragmented heterochrmatin of bronchial epithelial cells indicates apoptosis. *P < 0.05 versus the nonventilated control mice with room air; **†** *P* < 0.05 versus all other groups. Scale bars represent 20 μm. FiO_2_ = fraction of inspired oxygen

Histological examinations and transmission electron microscopy indicated that the animal lung injured by high-tidal-volume mechanical ventilation with hyperoxia displayed a hemorrhaging pattern, severe congestion, enlargement, neutrophil sequestration, and disruption of the airway ultrastructure: increased of secretory vesicles and epithelial apoptosis characterized by nuclear condensation (Fig. 6a, b). The lung injury score quantification confirmed that mechanical stretch induced severe lung damage during hyperoxia and the lung damage was substantially attenuated by treatment with SIS3 or in Src-deficient mice (Fig. 6c). Moreover, Src-deficient mice and pharmacologic inhibition with SIS3 improved the decreases of the gas exchange (PaO_2_/FiO_2_ ratio) in mice receiving a tidal volume of 30 mL/kg with hyperoxia (Fig. 6d).

## Discussion

Mechanical ventilation and concurrent hyperoxia, in the early and late fibroproliferative phases of ALI, is often required to prevent hypoxemia in patients with ARDS [1, 30]. Even while using the protective ventilatory strategy, pathologic lung over-distension may occur in the more compliant lung in patients with ARDS. Moreover, hyperoxia can mediate ALI characterized by disruption of the alveolar-capillary barrier, enhanced diffuse lung inflammation, pulmonary edema, and loss of lung compliance [4–6, 8, 14]. Although some recent improvements have been made in the prevention of VILI, it remains a crucial clinical problem, and the pathogenetic mechanisms must be further defined [14, 31]. Previous studies of murine model of VILI and endoplasmic reticulum stress in alveolar epithelial cells have shown the crucial roles of Src, Smad3, and MMP-9 pathways in the regulation of epithelial-mesenchymal transition [15, 20, 32]. Additionally, recent studies employing alveolar type II epithelial cells and rats have exhibited that cyclic mechanical stretch can induce ALI by activating the TGF-β/Smad pathway and inducing oxidative stress and MMP-9 production [5, 9, 13]. In the current mouse model of VILI, we demonstrated that high-tidal-volume ventilation plus hyperoxia further activated Src and Smad3 signaling pathways and exacerbated lung damage. Furthermore, pharmacologic inhibition with SIS3 and Src-deficient mice attenuated microvascular leak, neutrophil accumulation, upregulation of Src and Smad3, and MIP-2 and MMP-9 production.

A previous rat study of ALI showed that hyperoxia increased high-tidal-volume -induced production of MIP-2, a functional homolog of human interleukin-8 that is related to neutrophil sequestration [4]. Neutrophils, mostly chemoattracted by MIP-2, are the predominant inflammatory cells involved in the process of VILI and are crucial for the generation of ROS [31, 33]. Previous studies have demonstrated that excessive ROS production in response to mechanical ventilation plus hyperoxia can act as direct cell toxins and as secondary messengers by inducing pulmonary epithelium to secrete chemoattractants and proinflammatory cytokines that causes an influx of neutrophils to the lung [5, 24, 33]. The potential sources of oxidant-generating enzymes include NADPH oxidase, xanthine oxidase, and nitric oxide synthase [14]. The NADPH oxidase, a multimeric enzyme complex comprising NOX1-5 and two larger dual oxidases, DUOX1 and DUOX2, has been implicated as a major source for increased superoxide production in response to mechanical ventilation in pulmonary epithelial cells [5, 8, 34]. A recent isolated and perfused rat lung model of VILI also showed that pharmacologic inhibition with apocycin, a NADPH oxidase inhibitor, reduced high-tidal-volume ventilation-induced NADPH oxidase activity and the production of ROS [11]. *In vitro*, Src protein tyrosine kinases can be activated by TGF-β1 through NADPH oxidase-dependent redox signaling in H358 cells, a small cell lung carcinoma cell line [35]. Recently, we have revealed that the up-regulation of NOX2 occurred in mice subjected to a tidal volume of 30 mL/kg with hyperoxia compared with those subjected to a tidal volume of 30 mL/kg with room air and the control mice. However, no substantial differences on NOX1 expression occurred between mice subjected to a tidal volume at 30 mL/kg with or without hyperoxia [8]. In the current study, we demonstrated that administering hyperoxia augmented stretch-induced neutrophil infiltration, MIP-2 production, MDA level, NOX2 expression, and NADPH oxidase activity, which can be suppressed by Src-deficient mice. Additionally, increased level of total antioxidant capacity in this murine model of VILI was observed after using Src-deficient mice.

MMP-9, a gelatinase B granule stored in the neutrophils, has been shown to participate in both collagen turnover and growth factor activation [9]. It is secreted by many cells, including neutrophils, macrophages, fibroblasts, and endothelial and epithelial cells. Importantly, MMPs are crucial in angiogenesis, tissue remodeling, the restoration of functional connective tissue in the wound-healing process [15]. The migration of activated neutrophils through the basement membrane is aided by digestion of alveolar epithelial ECM by MMP-9, which is considered crucial in the pathogenesis of ALI [15, 36]. Previous rat models of VILI have revealed that pharmacologic inhibition with CMT-3 or doxycycline attenuated high-tidal-volume mechanical ventilation-induced neutrophil accumulation, MMP-9 activation, and lung injury [9, 10]. It is known that Src or Smad3 can activate the promoter of MMP-9 indirectly by p300/nuclear factor-κB (NF-κB) binding or forming a complex with activator protein-1 [37, 38]. Moreover, a previous murine study of hyperoxia-mediated ALI also demonstrated that serine/threonine kinase/protein kinase B (Akt) and NF-κB regulated MPO activity, lipid peroxidation, and the releases of MIP-2 and MMP-9 [16, 17, 24]. We observed that hyperoxia mediated lung damage by generating free radicals. Additionally, we found that mice exposed to hyperoxia would be more susceptible to the deleterious effects of high-tidal-volume mechanical ventilation through upregulation of NOX2, MMP-9 mRNA expression, production of MIP-2 and MMP-9, and influx of neutrophils.

Lipopolysaccharide-induced and ischemia-reperfusion-mediated ALI in rodents have revealed pharmacologic inhibition of Src phosphorylation by Src inhibitors suppressed neutrophil influx, upregulated MMP-9 mRNA expression, and MIP-2 production [21, 23]. Furthermore, a previous murine model of endotoxin-induced ALI also demonstrated that Src activation was crucial in the regulation of NF-κB and α_v_β_3_ integrin signaling [22]. Src-mediated activation of NADPH oxidase and superoxide production was related to phosphorylation of p47^*phox*^ tyrosine, a major cytosolic subunits of NADPH oxidase in lung endothelial cells [24]. These studies suggest that Src protein tyrosine kinase is a major regulator for the production of chemotactic factor, recruitment of neutrophils, and NADPH oxidase activation in ALI [12, 21–24]. Recently, mechanical stretch-induced activation of Src in rats was proven to increase intracellular signal transduction and regulate the expression of cell junction proteins [7]. Src activation may promote the degradation of junctional proteins from their cytoskeletal anchors and cause endothelial gap formation, resulting in an increase in vascular permeability [7]. Inhibition of Src phosphorylation is crucial in reducing cyclic stretching-induced lung injury, enhances barrier function, and attenuates intercellular signaling [7]. Collectively, high-tidal-volume mechanical ventilation can modulate Src phosphorylation by activating Ca^2+^ entry through focal adhesion kinases, G protein-coupled receptors, integrin receptors, and stretch-activated cation channels [2]. Our results demonstrated that mechanical ventilation with hyperoxia further induced oxidative stress, MMP-9 expression, and the phosphorylation of Src in murine lung tissue, which can be suppressed in Src-deficient mice. We further explored the roles of Src activation and its downstream signaling targets in the regulation of hyperoxia-augmented VILI.

**Fig. 7:**
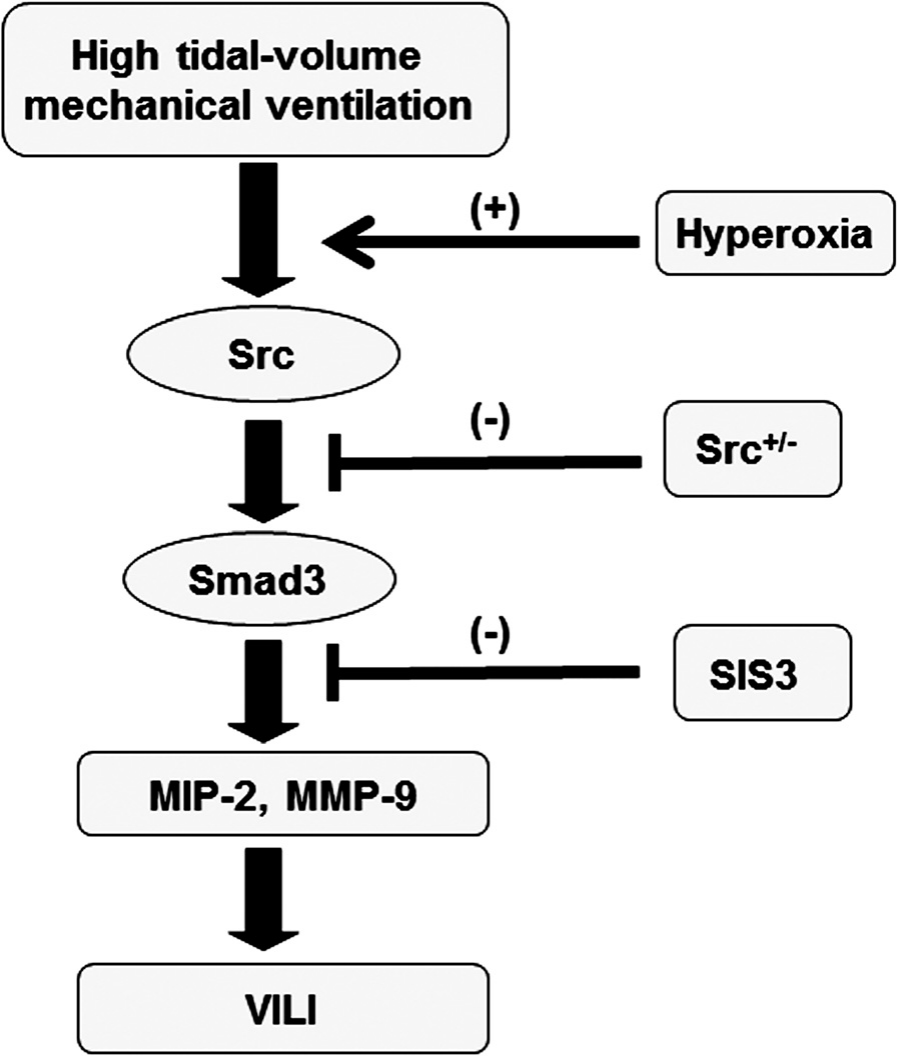
Schematic figure illustrating the signaling pathway activation with high-tidal-volume mechanical ventilation and hyperoxia. Hyperoxia-induced augmentation of mechanical stretch-mediated cytokine production and lung injury were attenuated with Src-deficient mice and pharmacological inhibition of Smad3 activity by SIS. MIP-2 = macrophage inflammatory protein-2; MMP-9 = matrix metalloproteinase-9; SIS3 = specific inhibitor of Smad3; Src^+/− =^ Src-deficient mice^;^ VILI = ventilator-induced lung injury

In addition to playing a role in TGF-β1-mediated lung fibrogenesis, Smad3, a cytoplasmic signal transducer protein, was proven to be involved in the pathogenesis of ALI [3, 13, 19, 25]. Activation of Smad-2/3 signaling is associated with increased neutrophil sequestration in a murine model of LPS-induced ALI [19]. Recent studies of LPS- or cecal ligation-induced ALI in rats have also demonstrated that Smad3 activation increases the level of malondialdehyde, an aldehydic secondary product of ROS, and induces the lung edema and ultrastructural damage of lung tissue [13, 25]. Moreover, oxidant stress is known to regulate tissue inflammation through activation of lipid peroxidation, protein peroxidation, DNA damage, and transcription factors such as NF-κB and Smad [37]. Once activated, receptor-regulated Smads (R-Smads, Smad2, and Smad3) become phophorylated and form heteromeric complexes with common Smads (Co-Smad, Smad4). These complexes translocate to the nucleus, where they bind to TGF-β-responsive promoter DNA, either directly through the Smad-binding elements or in conjunction with other co-activators, and upregulate MMP-9 expression [37]. Further, Src protein kinases can be activated by TGF-β and TGF-β-stimulated Src expression sustained the duration of Smad3 phosphorylation in vascular smooth muscle cells [39]. However, the mechanisms by which Src regulates Smad3 in hyperoxia-augmented ALI are not well defined. Our data indicated that Src activation and Src-dependent phosphorylation of Smad3 were involved in the regulation of NADPH oxidase, intracellular ROS generation, and MMP-9 production in a mouse model of VILI. Furthermore, we observed that high-tidal-volume ventilation and hyperoxia enhanced the expression of Smad3, which was reduced by Src-deficient mice, suggesting that Src was upstream of Smad3 signaling pathway (Fig. 7).

This study had some limitations. There are other potential upstream signal molecules involved in the regulation of hyperoxia-augmented MMP-9 production. An *in vitro* study of human tracheal smooth muscle cells revealed that TNF-α-induced MMP-9 expression was regulated through phosphoinositide 3-OH kinase and Akt phosphorylation [38]. Recent studies used murine models of hyperoxia-mediated ALI have also demonstrated that inhibition of DNA-binding activity of NF-κB reduced lung injury and MMP-9 production [22, 36]. Two time points for measurements were used based on our previous findings that upstream regulators occurs early in VILI and changes of lung microvascular permeability occurs later [8, 27]. Additional experiments are necessary to explore potential regulators of VILI with hyperoxia.

## Conclusions

Using our *in vivo* mouse model of ALI, we have demonstrated that administration of hyperoxia aggravated high-tidal-volume ventilation-induced destruction of the alveolar-capillary barrier, resulting in increased lung microvascular permeability, neutrophil influx, oxidative stress, and MIP-2 and MMP-9 production. The injurious process caused by mechanical ventilation with or without hyperoxia partly depends on the activation of Src and Smad3. Knowledge of the effect of mechanical forces on Src and Smad3 signaling pathways allows clarification of the molecular and cellular mechanisms regulating acute exudative phase of ARDS. Therefore, inhibiting Src and Smad3 might serve as a novel therapeutic target in the management of mechanically ventilated patients with ARDS exposed to hyperoxia, whose mortality has remained substantially high with the present lung-protective ventilation strategy.

## Abbreviations

ALI: Acute lung injury
ARDS: Acute respiratory distress syndrome
ECM: Extracellular matrix
EBD: Evans blue dye
ES: Embryonic stem
MDA: Malondialdehyde
MIP-2: Macrophage inflammatory protein-2
MMP-9: Matrix metalloproteinase-9
MPO: Myeloperoxidase
NADPH: Nicotinamide adenine dinucleotide phosphate
NF-κB: Nuclear factor-κB
NOX: NADPH oxidase
ROS: Reactive oxygen species
PCR: Polymerase chain reaction
PI3K: Phosphoinositide 3-OH kinase
SIS3: Specific inhibitor of Smad3
TGF-β1: Transforming growth factor-β1
TNF-α: Tumor necrosis factor-α
VILI: Ventilator-induced lung injury
V_T_: Tidal volume

## References

[1] Matthay MA, Zimmerman GA, Esmon C, Bhattacharya J, Coller B, Doerschuk CM (2003). Future research directions in acute lung injury: summary of a National Heart, Lung, and Blood Institute working group. Am J Respir Crit Care Med.

[2] Miyahara T, Hamanaka K, Weber DS, Drake DA, Anghelescu M, Parker JC (2007). Phosphoinositide 3-kinase, Src, and Akt modulate acute ventilation-induced vascular permeability increases in mouse lungs. Am J Physiol Lung Cell Mol Physiol.

[3] Fahy RJ, Lichtenberger F, McKeegan CB, Nuovo GJ, Marsh CB, Wewers MD (2003). The acute respiratory distress syndrome: a role for transforming growth factor-beta 1. Am J Respir Cell Mol Biol.

[4] Quinn DA, Moufarrej RK, Volokhov A, Hales CA (2002). Interactions of lung stretch, hyperoxia, and MIP-2 production in ventilator-induced lung injury. J Appl Physiol (1985).

[5] Chapman KE, Sinclair SE, Zhuang D, Hassid A, Desai LP, Waters CM (2005). Cyclic mechanical strain increases reactive oxygen species production in pulmonary epithelial cells. Am J Physiol Lung Cell Mol Physiol.

[6] Li LF, Yang CT, Huang CC, Liu YY, Kao KC, Lin HC (2011). Low-molecular-weight heparin reduces hyperoxia-augmented ventilator-induced lung injury via serine/threonine kinase-protein kinase B. Respir Res.

[7] Zhao T, Liu M, Gu C, Wang X, Wang Y (2014). Activation of c-Src tyrosine kinase mediated the degradation of occludin in ventilator-induced lung injury. Respir Res.

[8] Liu YY, Li LF, Fu JY, Kao KC, Huang CC, Chien Y (2014). Induced pluripotent stem cell therapy ameliorates hyperoxia-augmented ventilator-induced lung injury through suppressing the Src pathway. PLoS One.

[9] Kim JH, Suk MH, Yoon DW, Lee SH, Hur GY, Jung KH (2006). Inhibition of matrix metalloproteinase-9 prevents neutrophilic inflammation in ventilator-induced lung injury. Am J Physiol Lung Cell Mol Physiol.

[10] Doroszko A, Hurst TS, Polewicz D, Sawicka J, Fert-Bober J, Johnson DH (2010). Effects of MMP-9 inhibition by doxycycline on proteome of lungs in high tidal volume mechanical ventilation-induced acute lung injury. Proteome Sci.

[11] Chiang CH, Chuang CH, Liu SL, Lee TS, Kou YR, Zhang H (2011). Apocynin attenuates ventilator-induced lung injury in an isolated and perfused rat lung model. Intensive Care Med.

[12] Okutani D, Lodyga M, Han B, Liu M (2006). Src protein tyrosine kinase family and acute inflammatory responses. Am J Physiol Lung Cell Mol Physiol.

[13] Mu E, Ding R, An X, Li X, Chen S, Ma X (2012). Heparin attenuates lipopolysaccharide-induced acute lung injury by inhibiting nitric oxide synthase and TGF-beta/Smad signaling pathway. Thromb Res.

[14] Kallet RH, Matthay MA (2013). Hyperoxic acute lung injury. Respir Care.

[15] Pelosi P, Rocco PR (2008). Effects of mechanical ventilation on the extracellular matrix. Intensive Care Med.

[16] Huang CH, Yang ML, Tsai CH, Li YC, Lin YJ, Kuan YH (2013). Ginkgo biloba leaves extract (EGb 761) attenuates lipopolysaccharide-induced acute lung injury via inhibition of oxidative stress and NF-kappaB-dependent matrix metalloproteinase-9 pathway. Phytomedicine.

[17] Chen WY, Huang YC, Yang ML, Lee CY, Chen CJ, Yeh CH (2014). Protective effect of rutin on LPS-induced acute lung injury via down-regulation of MIP-2 expression and MMP-9 activation through inhibition of Akt phosphorylation. Int Immunopharmacol.

[18] Foda HD, Rollo EE, Drews M, Conner C, Appelt K, Shalinsky DR (2001). Ventilator-induced lung injury upregulates and activates gelatinases and EMMPRIN: attenuation by the synthetic matrix metalloproteinase inhibitor, Prinomastat (AG3340). Am J Respir Cell Mol Biol.

[19] Fujino N, Kubo H, Suzuki T, He M, Suzuki T, Yamada M (2012). Administration of a specific inhibitor of neutrophil elastase attenuates pulmonary fibrosis after acute lung injury in mice. Exp Lung Res.

[20] Tanjore H, Cheng DS, Degryse AL, Zoz DF, Abdolrasulnia R, Lawson WE (2011). Alveolar epithelial cells undergo epithelial-to-mesenchymal transition in response to endoplasmic reticulum stress. J Biol Chem.

[21] Oyaizu T, Fung SY, Shiozaki A, Guan Z, Zhang Q, dos Santos CC (2012). Src tyrosine kinase inhibition prevents pulmonary ischemia-reperfusion-induced acute lung injury. Intensive Care Med.

[22] Lee HS, Moon C, Lee HW, Park EM, Cho MS, Kang JL (2007). Src tyrosine kinases mediate activations of NF-kappaB and integrin signal during lipopolysaccharide-induced acute lung injury. J Immunol.

[23] Severgnini M, Takahashi S, Tu P, Perides G, Homer RJ, Jhung JW (2005). Inhibition of the Src and Jak kinases protects against lipopolysaccharide-induced acute lung injury. Am J Respir Crit Care Med.

[24] Chowdhury AK, Watkins T, Parinandi NL, Saatian B, Kleinberg ME, Usatyuk PV (2005). Src-mediated tyrosine phosphorylation of p47phox in hyperoxia-induced activation of NADPH oxidase and generation of reactive oxygen species in lung endothelial cells. J Biol Chem.

[25] Xu F, Lin SH, Yang YZ, Guo R, Cao J, Liu Q (2013). The effect of curcumin on sepsis-induced acute lung injury in a rat model through the inhibition of the TGF-beta1/SMAD3 pathway. Int Immunopharmacol.

[26] Soriano P, Montgomery C, Geske R, Bradley A (1991). Targeted disruption of the c-src proto-oncogene leads to osteopetrosis in mice. Cell.

[27] Li LF, Liao SK, Ko YS, Lee CH, Quinn DA (2007). Hyperoxia increases ventilator-induced lung injury via mitogen-activated protein kinases: a prospective, controlled animal experiment. Crit Care.

[28] Jinnin M, Ihn H, Tamaki K (2006). Characterization of SIS3, a novel specific inhibitor of Smad3, and its effect on transforming growth factor-beta1-induced extracellular matrix expression. Mol Pharmacol.

[29] Ma B, Zhou PY, Ni W, Wei W, Ben DF, Lu W (2013). Inhibition of activin receptor-like kinase 5 induces matrix metallopeptidase 9 expression and aggravates lipopolysaccharide-induced pulmonary injury in mice. Eur Rev Med Pharmacol Sci.

[30] ARDSNet (2000). Ventilation with lower tidal volumes as compared with traditional tidal volumes for acute lung injury and the acute respiratory distress syndrome. The Acute Respiratory Distress Syndrome Network. N Engl J Med.

[31] Park HS, Kim SR, Lee YC (2009). Impact of oxidative stress on lung diseases. Respirology.

[32] Li LF, Liu YY, Kao KC, Wu CT, Chang CH, Hung CY (2014). Mechanical ventilation augments bleomycin-induced epithelial-mesenchymal transition through the Src pathway. Lab Invest.

[33] Syrkina O, Jafari B, Hales CA, Quinn DA (2008). Oxidant stress mediates inflammation and apoptosis in ventilator-induced lung injury. Respirology.

[34] Lee IT, Yang CM (2012). Role of NADPH oxidase/ROS in pro-inflammatory mediators-induced airway and pulmonary diseases. Biochem Pharmacol.

[35] Zhang H, Davies KJ, Forman HJ (2015). TGFbeta1 rapidly activates Src through a non-canonical redox signaling mechanism. Arch Biochem Biophys.

[36] Tao W, Shu YS, Miao QB, Zhu YB (2012). Attenuation of hyperoxia-induced lung injury in rats by adrenomedullin. Inflammation.

[37] Gordon GM, Ledee DR, Feuer WJ, Fini ME (2009). Cytokines and signaling pathways regulating matrix metalloproteinase-9 (MMP-9) expression in corneal epithelial cells. J Cell Physiol.

[38] Lee CW, Lin CC, Lin WN, Liang KC, Luo SF, Wu CB (2007). TNF-alpha induces MMP-9 expression via activation of Src/EGFR, PDGFR/PI3K/Akt cascade and promotion of NF-kappaB/p300 binding in human tracheal smooth muscle cells. Am J Physiol Lung Cell Mol Physiol.

[39] Samarakoon R, Chitnis SS, Higgins SP, Higgins CE, Krepinsky JC, Higgins PJ (2011). Redox-induced Src kinase and caveolin-1 signaling in TGF-beta1-initiated SMAD2/3 activation and PAI-1 expression. PLoS One.

